# Water Cloud Detection with Circular Polarization Lidar: A Semianalytic Monte Carlo Simulation Approach

**DOI:** 10.3390/s22041679

**Published:** 2022-02-21

**Authors:** Wiqas Ahmad, Kai Zhang, Yicheng Tong, Da Xiao, Lingyun Wu, Dong Liu

**Affiliations:** International Research Center for Advanced Photonics, State Key Laboratory of Modern Optical Instrumentation, College of Optical Science and Engineering, Zhejiang University, Hangzhou 310027, China; wiqas@zju.edu.cn (W.A.); 3150104660@zju.edu.cn (K.Z.); yichengtong@zju.edu.cn (Y.T.); 426196@zju.edu.cn (D.X.); wlyun@zju.edu.cn (L.W.)

**Keywords:** linear and circular polarization lidars, water clouds, Monte Carlo simulation, linear and circular depolarization ratios, Mishchenko–Hovenier relationship

## Abstract

This work presents polarization property studies of water clouds using a circular polarization lidar through a simulation approach. The simulation approach is based on a polarized, semianalytic Monte Carlo method under multiple-scattering conditions and considers three types of water clouds (namely homogeneous, inhomogeneous and partially inhomogeneous). The simulation results indicate that the layer-integrated circular depolarization ratios show similar variation trends as those of layer-integrated linear depolarization ratios. The Mishchenko–Hovenier relationship is validated to correlate the simulated layer-integrated circular and linear depolarization ratios. In addition, the cloud droplet effective radius, extinction coefficient, lidar field-of-view (FOV) and height of the cloud bottom are all found to affect the layer-integrated depolarization ratio. The current work theoretically indicates that a circular polarization lidar can efficiently perform measurements of water clouds, enjoying the advantage of higher sensitivity compared to a traditional linear polarization lidar. Hence, it should be of interest to researchers in fields of polarization lidar applications.

## 1. Introduction

In the early 1950s, the concept of polarization was first exploited in the microwave radar to improve its signal performance [1]. Various efforts were then conducted to develop a theoretical model of polarization expression using a scattering matrix [2,3]. Later, after the invention of the laser in the 1960s, these polarization techniques were employed for the measurement of atmospheric lidar [4,5]. In early attempts, the laser depolarization ratio from the non-spherical particles (larger than the incident wavelength) was found to be much higher by comparison than the radar depolarization ratio (particles smaller than the incident wavelength) [6]. Such attempts provided evidence that the polarization lidar has an eminent future in atmospheric exploration. Extensive literature on polarization lidars was then published by [7,8,9].

Generally, the observation of a linear polarization lidar is based on measuring the depolarization ratio between the two receiving channels [10,11]. The depolarization ratio reveals the microphysical properties of water clouds and has a long history in atmospheric research [12,13,14,15]. It has been proven to be very effective parameter for discriminating water clouds from ice clouds, thereby discriminating spherical from non-spherical particles [16,17]. Theoretically, it is based on the calculation of the scattering matrix, whose elements are symmetric for backscattering from spherical particles and asymmetric for backscattering from non-spherical particles [18]. Thus, backscattering from water clouds composed of spherical droplets does not cause any depolarization assuming single-scattering. Depolarization is caused either by clouds composed of ice crystals in the case of both single-scattering and multiple-scattering or even composed of spherical water droplets in the case of multiple-scattering alone. In order to investigate the effects of multiple-scattering on the depolarization ratio of water clouds, various experimental and theoretical works have been carried out so far [19,20,21,22].

A theoretical study based on Monte Carlo simulation on cloud phase discrimination using a circular polarization lidar is presented by [23]. The author found that spherical and non-spherical particles can be discriminated by measuring the magnitude as well as the rotation direction of the circularly polarized component of the backscattered light. Furthermore, he found that the circular polarization lidar technique for cloud phase discrimination is less sensitive to multiple-scattering. The authors in [24] reported that the circular polarization lidar does not show azimuthal patterns in the lidar backscattering signal, while the linear polarization lidar does in the case of spherical particles. However, the perpendicular backscattering signal component of the circular polarization lidar is less prone to noise, and therefore, its signal to noise ratio is two to five times higher than that of linear polarization lidar [24]. Several studies have been conducted measuring the depolarization ratio in the backscattering signal of a circular polarization lidar [25,26]. However, complete guidance about the measurement of the circular depolarization ratio from water clouds is still lacking.

A more sophisticated tool to model the multiple-scattering effects in the backscatter lidar signal from water clouds is the use of Monte Carlo (MC) simulation, which was first employed by [27,28,29]. A linear polarization lidar is always used as a key parameter for atmospheric detection [30]. Limited work has been published highlighting the possible use of a circular polarization lidar [23,26]. In 1947, circular polarization was used for the first time to increase the contrast detection in a radar backscattering signal [31]. In the 1960s, by conducting experiments underwater, Ref. [32] showed that visibility can be improved by reducing the backscattered light using the circular polarization technique under the condition of small hydrosols. In recent years, the authors in [33] showed that a circularly polarized laser beam backscattered from water fog produces less contrast in the perpendicular channel of the lidar as compared to linear polarized beam. Furthermore, they found a circularly polarized beam depolarizes much faster than a linear polarized beam. Water cloud droplets can also cause circular depolarization at a scattering angle less than 180° and increases more rapidly as we move away from perfect backscattering with increasing droplet size [25]. Thus, further research is required to explore more about the possible use of circular polarization lidars in detecting water clouds and cannot be ignored.

In this article, we present a more in-depth study through simulation, showing a great deal of variety in water clouds using a circular polarization lidar. We use the incident circularly polarized beam to calculate the depolarization ratio of homogeneous, inhomogeneous and partially inhomogeneous clouds. In each cloud type, five different cloud cases are considered, and for each cloud case, a simulation is carried out separately. The simulation is carried out using the polarized semianalytic Monte Carlo (PSMC) method, whose convergence scheme is much faster compared to the standard Monte Carlo method. The working scheme of the PSMC method is given is Section 4. Scattered Stokes vectors are obtained from the simulation results under a multiple-scattering regime, and the layer-integrated backscattered signal components, parallel and perpendicular, are computed. From these signal components, the layer-integrated circular depolarization ratios are obtained for five different cloud cases considered in each homogeneous, inhomogeneous and partially inhomogeneous water cloud. The simulation is repeated for the incident linearly polarized beam to obtain the layer-integrated linear depolarization ratio in order to make comparisons.

Note that for the simulation, only two types of incident laser beams are considered, linearly, parallelly and left-handed, circularly polarized. The remaining two types of laser beams, linearly, perpendicularly and right-handed, circularly polarized, are simply ignored because they are equivalent to the aforementioned two types in the theoretical aspect and yield the same results from our corresponding simulation. Finally, an important “Mishchenko–Hovenier relationship” is presented and validated through our simulated data. The main structure of the paper is as follows: cloud models and methods are presented is Section 2. The theoretical framework of the paper is described in Section 3. The polarized semianalytic Monte Carlo (PSMC) method is presented in Section 4. The important Mishchenko–Hovenier relationship is discussed in Section 5. Section 6 comprises the main discussion of the paper. Finally, the conclusion of the paper is highlighted in Section 7.

## 2. Cloud Models and Methods

The basic microphysical properties characterizing a water cloud are the cloud droplet effective radius $R_{e}$, the cloud extinction coefficient $\alpha_{e}$ (single-scattering), the cloud droplet number concentration $N_{d}$ and the liquid water content LWC [34]. Generally, water clouds are composed of micro-liquid droplets almost spherical in shape. However, some external factors, for instance force of gravity, might influence their spherical symmetry [35]. The variation in the properties of clean air both in time and space also affects cloud droplets. Therefore, the cloud droplet sizes cannot be exactly known. Hence, the radius r of a droplet should be chosen by selecting random numbers, and the size per unit volume is described by cloud droplet size distribution (DSD).

The water cloud DSD can be best modeled with gamma distribution [36]. Here, we would like to follow the single-mode modified gamma distribution presented by [37] as:

(1) $$n(r)=\frac{N_{d}}{R_{m}}\cdot \frac{1}{(\gamma -1)!}\cdot {\left(\frac{r}{R_{m}}\right)}^{\gamma -1}\cdot \exp(-\frac{r}{R_{m}}),$$

where $R_{m}$ is the so-called mode radius, $N_{d}$ is the total number of droplets (cm^−3^) and γ is the shape parameter with value γ=7 for the C1-cloud model [35]. The water cloud DSD is expressed in units of $(m^{-3}\mu m^{-1}).$ After some manipulation [34], we can derive the cloud effective radius $R_{e}$ from Equation (1) as:

(2) $$R_{e}=R_{m}(\gamma +2).$$

**Figure 1:**
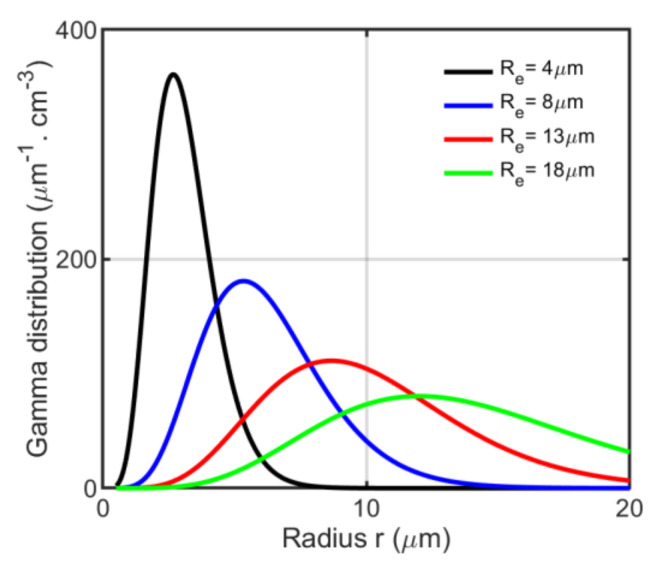
The gamma droplet size distribution with four different effective radii, i.e., $R_{e}=4,8,13,18\;\mu m$ with a shape parameter γ=7.

The cloud droplet effective radius $R_{e}$ of a typical water cloud mainly ranges from 4 to 20 μm [5]. The curves of gamma DSD for four cloud droplet effective radii $R_{e}$ are plotted in Figure 1. The values of extinction coefficient $\alpha_{e}$$\left(m^{-1}\right)$ can be assumed. Then, we can calculate the liquid water content LWC as [34]:

(3) $$LWC=\frac{2}{3}\rho_{w}\alpha_{e}R_{e},$$

where $\rho_{w}$ is the density of water. LWC is expressed in units of $gm^{-3}$. The cloud droplet concentration number $N_{d}$ is given by [34]

(4) $$N_{d}=\frac{1}{2\pi }\frac{\alpha_{e}}{kR_{e}^{2}}.$$

where the parameter k is defined by [38] as the ratio of the cubic power of the volume mean droplet radius $R_{v}$ to the cubic power of the measured effective radius $R_{e}$, given by:

(5) $$k=\frac{R_{v}^{3}}{R_{e}^{3}}.$$

In order to perform the simulation, we modeled three types of water clouds with a cloud simulator, mainly homogeneous, inhomogeneous and partially inhomogeneous, considering five different cloud cases in each as shown in Table 1. Note that all our cloud models are two-dimensional, which means that the cloud properties vary along the vertical direction and remain constant along the horizontal direction. It is one of the features of our PSMC. In homogeneous water clouds, we kept both the cloud droplet effective radius $R_{e}$ and extinction coefficient $\alpha_{e}$ constant throughout the cloud from the bottom to the top. Therefore, the cloud as a whole can be considered as a single cloud layer. In inhomogeneous water clouds, both the cloud droplet effective radius $R_{e}$ and extinction coefficient $\alpha_{e}$ are changed in a sequence from low values at the bottom to high values at the top, such that each layer has relatively different values of $R_{e}$, and $\alpha_{e}$ ranges from the bottom to the top of the cloud. In partial homogeneous water clouds, the cloud effective radius $R_{e}$ is kept constant and the extinction coefficient $\alpha_{e}$ of each layer is varied throughout the cloud.

For each cloud case, the simulation is repeated two times. The first one is carried out using the incident left-handed, circularly polarized beam and the second is carried out using the incident linearly, parallelly polarized beam. Each simulation takes about five to six hours to complete, the speed of which depends on the values of input parameters as well as the computer processor. The geometrical depth of the cloud is kept constant for each cloud case. The FOV of the lidar for each cloud case is kept at 1 mrad except the cloud Case2, for which the FOV is 2 mrad. The number of photons launched is $10^{9}$. The maximum number of scatterings in each layer are kept at 7. The number of averaging signal layers is 30. The spatial resolution is 5 m, which can be obtained by dividing the total cloud geometrical depth (150 m) by the total signal layers (30). The simulator also requires the scattering-phase function for each cloud droplet effective radius in a separate file. The file represents the scattering-phase matrix containing all the elements of the scattering-phase matrix with associated scattering angles calculated by Mie theory.

Mie theory provides the mathematical solution of scattering from a spherical particle with a size comparable to the incident wavelength, first presented by Gustav Mie in 1908 [39]. The theory is very complex and requires cumbersome mathematics; however, a comprehensive discussion can be found in [36,40]. The droplets in water clouds are generally spherical, and hence, Mie theory can be applied to find out all the elements of the scattering-phase matrix. These elements are obtained using the incident 532 nm laser wavelength. The simulation reproduces the scattered Stokes parameters via the PSMC approach by utilizing the polarized scattering-phase function along with the cloud properties and features of lidar as inputs.

## 3. Mueller–Stokes Formalism

A polarization lidar is either based on linear or circular polarization [24]. In general, the polarization state of light is fully described by the four Stokes parameters, I,Q,U,V, combined into one vector known as the Stokes vector [41]. According to Mueller–Stokes formalism, the scattering of an incident polarized light with a Stokes vector $S_{0}=[I_{0},Q_{0},U_{0},V_{0}]$, from an infinitesimal scattering volume of a homogeneous water cloud with spherical cloud droplets distributed randomly can be described by the following equation [23]:

(6) $$\left[\begin{array}{c} I\\ Q\\ U\\ V \end{array}\right]=\left[\begin{array}{cccc} P_{11} & P_{12} & 0 & 0\\ P_{12} & P_{22} & 0 & 0\\ 0 & 0 & P_{33} & P_{34}\\ 0 & 0 & -P_{34} & P_{44} \end{array}\right]\left[\begin{array}{c} I_{0}\\ Q_{0}\\ U_{0}\\ V_{0} \end{array}\right],$$

where [I,Q,U,V] are the Stokes parameters of the scattered Stokes vector S describing the final state of polarization of the scattered light, and $P_{ij}$ are the elements of the scattering-phase matrix describing the scattering properties of water clouds that can be determined from Mie theory for spherical droplets, whereas i,j=1,2,3,4 [36,42,43]. The scattering-phase matrix $P_{ij}$ is related to the scattering amplitudes of the cloud droplets [44].

The direction of the scattered Stokes vector S in each scattering event is described with respect to the scattering plane. The scattering plane contains the direction vectors of the incident and scattered beams. This plane acts as a reference plane and changes its direction with each scattering in a multiple-scattering medium. In general, the scattered Stokes vector S is different from the one previously subjected to the next scattering. Thus, four components of the Stokes vector are required to track the polarization states of a photon that undergoes multiple-scattering [45]. The distribution of photons’ energy can be estimated from the scattering-phase function. Using the elements of the scattering-phase matrix along with the incident Stokes parameters, we can determine the scattering-phase function of a water cloud droplet for both unpolarized and polarized light.

### 3.1. The Scattering-Phase Function

The probability of scattered light with a certain scattering angle made with incident direction is given by the scattering-phase function. The scattering-phase function of water clouds for both polarized and unpolarized light can be calculated from Mie theory, as the droplets of water cloud are spherical. It is a function of the refractive index and size of the droplet and does not depend on their concentration number [43]. Generally, the single-scattering-phase function P(α,β) for a light incident on a spherical water cloud droplet with a general incident Stokes vector $S_{0}=[I_{0},Q_{0},U_{0},V_{0}]$ is written as [46]:

(7) $$P(\alpha ,\beta )=P_{11}(\alpha )I_{0}+P_{12}(\alpha )\left[Q_{0}\cos(2\beta )+U_{0}\sin(2\beta )\right].$$

$P_{11}\;\mathrm{and}\;P_{12}$ are the elements of the scattering-phase matrix, and α and β are the angles of scattering and rotation.

The scattering matrix M(α) expresses the scattering properties of the cloud droplet and symmetrical for spherical geometry. It is given by:

(8) $$M(\alpha )=\left[\begin{array}{cccc} P_{11}(\alpha ) & P_{12}(\alpha ) & 0 & 0\\ P_{12}(\alpha ) & P_{11}(\alpha ) & 0 & 0\\ 0 & 0 & P_{33}(\alpha ) & P_{34}(\alpha )\\ 0 & 0 & -P_{34}(\alpha ) & P_{33}(\alpha ) \end{array}\right].$$

The elements $P_{11},P_{12},P_{33}\;\mathrm{and}\;P_{34}$ (the dependency factor α is omitted for clarity) are related to the scattering amplitudes $S_{1}\;\mathrm{and}\;S_{2}$:

(9) $$\begin{array}{l} P_{11}=\frac{1}{2}\left({\left|S_{2}\right|}^{2}+{\left|S_{1}\right|}^{2}\right),\\ P_{12}=\frac{1}{2}\left({\left|S_{2}\right|}^{2}-{\left|S_{1}\right|}^{2}\right),\\ P_{33}=\frac{1}{2}\left(S_{2}{}^{*}S_{1}+S_{2}S_{1}{}^{*}\right),\\ P_{34}=-\frac{i}{2}\left(S_{1}S_{2}{}^{*}-S_{2}S_{1}{}^{*}\right). \end{array}$$

$S_{1}\;\mathrm{and}\;S_{2}$ depend on the size parameter, $x=2\pi R_{e}/\lambda$, where $R_{e}$ is the cloud droplet effective radius, the complex index of refraction of the particle m, the Riccati–Bessel functions, the spherical Bessel functions and the spherical Henkel functions. A more in-depth explanation of these parameters can be found in reference [43]. In our PSMC program, $S_{1}\;\mathrm{and}\;S_{2}$ are obtained from a Mie theory for every scattering angle α.

The scattering-phase function shows different properties for different incident polarized beams. For example, for an incident linearly polarized beam, the phase function is asymmetric about the incident axis because it depends on the angle β, whereas for an incident circularly polarized beam, the beam has no $Q_{0}\;\mathrm{and}\;U_{0}$ components, and thus, the phase function is symmetric about the axis [47]. The above phase function is valid for the scattering of both polarized and unpolarized beams. The scattering-phase function for unpolarized light is only a function of scattering angle α, i.e., $P(\alpha )=P_{11}(\alpha )$. Some of the characteristics of the scattering-phase function of water clouds in the visible range of electromagnetic spectrum are: (1) the scattering peaks at forward and backward angles are asymmetric; (2) despite the fact that the phase function strongly depends on size parameter, the dependency is weaker in the range of scattering $20^{\circ }-60^{\circ }$; (3) forward–scattering peaks are stronger; (4) the size parameter *x* can be derived from scattering at three angles, $\alpha =0,\;\alpha_{r},\;\pi$, where $\alpha_{r}$ is approximately 138°, indicating the enhanced scattering near rainbow [35].

**Figure 2:**
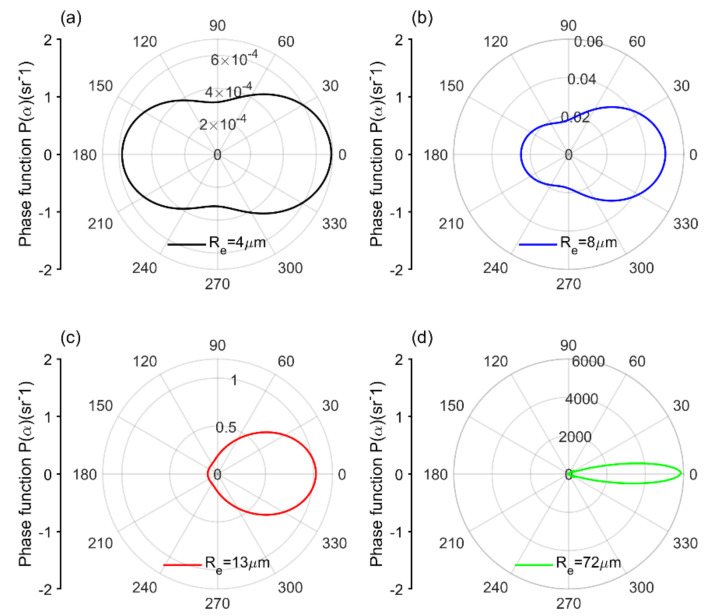
The polar representation of the scattering-phase function calculated for four water cloud droplet size distributions by Mie theory. As the size parameter of the cloud droplet increases from (**a**–**d**), the scattering at forward angles become more prominent and the forward–scattering peak becomes narrower.

The scattering-phase function of water cloud is plotted on a polar plot, as shown in Figure 2. The scattering intensity varies with the size of the cloud droplet. The forward peak of the scattering-phase function can be explained with Mie theory. It has a width that varies inversely with the droplet size and, consequently, larger droplets scatter the incident light with a small, solid angle in comparison to smaller droplets, which is evident from the figure [48]. We intentionally increase the size of the droplet to $R_{e}=72\;\mu m$ in order to resolve the forward scattering peak; otherwise, the real $R_{e}$ is comparatively much smaller in size. The backward to forward scattering ratio is related to the asymmetry factor g defined as the integral over all solid angles by P(α)cosα [49,50]. For isotropic-scattering, g=0, whereas for forward- and backward-scattering, g>0 and g<0.

### 3.2. Linearly Polarized Beam

The Stokes vector for an incident linearly, parallelly polarized beam is written as:

(10) $$S_{0}=\left[1\;1\;0\;0\right].$$

For a linearly, perpendicularly polarized beam, it becomes

(11) $$S_{0}=\left[1\;-1\;0\;0\right].$$

In the case of spherical water cloud droplets which perfectly backscatter light at a scattering angle of 180°, the scattering-phase matrix element $P_{12}$ is zero, i.e., $P_{12}=0$ [23].

Thus, the phase function of Equation (7) in this case is just a function of scattering angle α, as:

(12) $$P(\alpha )=P_{11}(\alpha ).$$

The theoretical linearly, parallelly and linearly, perpendicularly polarized scattering-phase functions relative to the incident beam polarization are given by [24]:

(13) $$P_{lin∥}\left(\alpha \right)=\frac{1}{8}\left(3L_{1}(\alpha )+3L_{2}(\alpha )\cos^{2}\alpha +2L_{3}(\alpha )\cos\alpha \right)$$

and

(14) $$P_{lin⊥}\left(\alpha \right)=\frac{1}{8}\left(L_{1}(\alpha )+L_{2}(\alpha )\cos^{2}\alpha -2L_{3}(\alpha )\cos\alpha \right).$$

The subscripts ∥, ⊥ indicate the parallelly and perpendicularly polarized components of the phase function relative to the incident beam polarization, and $L_{1},L_{2}\;\mathrm{and}\;L_{3}$ are related as:

(15) $$L_{1}=\frac{P_{11}-P_{12}}{2},L_{2}=\frac{P_{11}+P_{12}}{2},L_{3}=\frac{P_{33}}{2}.$$

**Figure 3:**
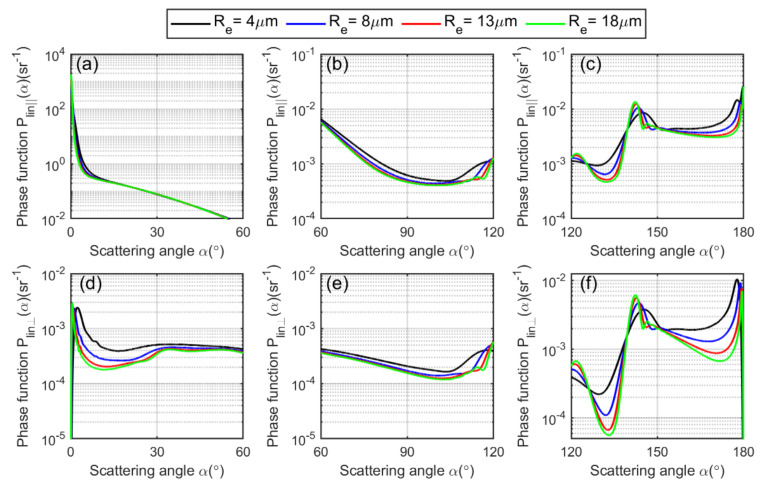
(**a**–**c**) show linearly, parallelly polarized scattering-phase function of a spherical cloud droplet plotted versus scattering angle calculated by using Equation (13). (**d**–**f**) show the linearly, perpendicularly polarized scattering-phase function of a spherical cloud droplet plotted versus scattering angle calculated using Equation (14). The scattering range at forward hemisphere is 0°–60°, at centered hemisphere it is 60°–120°, and at backward hemisphere it is 120°–180°.

The $P_{lin∥}(\alpha )\;\mathrm{and}\;P_{lin⊥}(\alpha )$ are plotted versus scattering angle, depicted in Figure 3.

**Figure 4:**
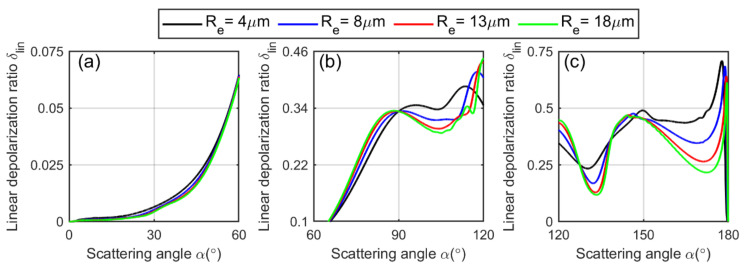
The theoretical linear depolarization ratio as a function of scattering angle of a single water cloud droplet (C1-cloud, γ=7,m=1.33) calculated by using Equation (16), (**a**) at forward hemisphere 0°–60°, (**b**) at centered hemisphere 60°–120° (**c**) and at backward hemisphere 120°–180°.

We separate the phase function into forward, centered and backward hemispheres. It is evident from the figure that the linearly polarized phase function depends on the cloud droplet effective radius $R_{e}$, as each curve varies with $R_{e}$ accordingly. The theoretical linear depolarization ratio as a function of scattering angle for a spherical water cloud droplet can be calculated by Mie theory as [51]:

(16) $$\delta_{lin}(x,m,z,\alpha )=\frac{L_{1}(\alpha )+L_{2}(\alpha )\cos^{2}\alpha -2L_{3}(\alpha )\cos\alpha }{3L_{1}(\alpha )+3L_{2}(\alpha )\cos^{2}\alpha +2L_{3}(\alpha )\cos\alpha },$$

with $L_{1},L_{2}\;\mathrm{and}\;L_{3}$ given by Equation (15). Here, $P_{11},P_{12}\;\mathrm{and}\;P_{33}$ are the elements of the scattering-phase matrix that can be obtained from Mie theory [36], x is the size parameter, m is the refractive index of water, z is the distance where the scattering occurs and α is the arbitrary scattering angle. It is plotted in Figure 4. Note that the calculation of Equation (16) is based on the double-scattering model, which is well explained in [24].

From the curves of the depolarization ratio in Figure 4, we can conclude that [52]:

- At the forward hemisphere 0°–60°, the depolarization ratio is almost negligible from $0^{\circ }$ to $20^{\circ }$ and then gradually increases.

- At the centered hemisphere $\left(60^{\circ }-120^{\circ }\right)$, the depolarization ratio increases in the beginning and then decreases before reaching the higher values.

- At the backward hemisphere $\left(120^{\circ }–180^{\circ }\right)$, the depolarization begins from certain values, experiencing two peaks and two valleys in a sequence before reaching the maximum close to 180°.

- The depolarization ratio is zero for the perfect backscattering at an angle of 180°, which takes place in the backward hemisphere.
- The maximum depolarization is produced in the vicinity of 180° in the backward hemisphere.
- Overall, the scattering in the backward hemisphere comparatively causes more depolarization.

### 3.3. Circularly Polarized Beam

When the incident laser beam is right-handed and circularly polarized, the Stokes vector is given by:

(17) $$S_{0}=\left[1\;0\;0\;1\right].$$

For a left-handed, circularly polarized beam, the Stokes vector is

(18) $$S_{0}=\left[1\;0\;0\;-1\right].$$

The positive and negative signs show the handedness of the circular polarization components, respectively [23]. The general phase function is expressed with the same expression as Equation (7). Similarly, the theoretical circularly, parallelly and circularly, perpendicularly polarized scattering-phase functions can be calculated as [24]:

(19) $$P_{cir∥}\left(\alpha \right)=\frac{1}{4}\left(L_{1}(\alpha )+L_{2}(\theta )\cos^{2}\alpha +2L_{3}(\alpha )\cos\alpha \right)$$

and

(20) $$P_{cir⊥}\left(\alpha \right)=\frac{1}{4}\left(L_{1}(\alpha )+L_{2}(\alpha )\cos^{2}\alpha -2L_{3}(\alpha )\cos\alpha \right).$$

**Figure 5:**
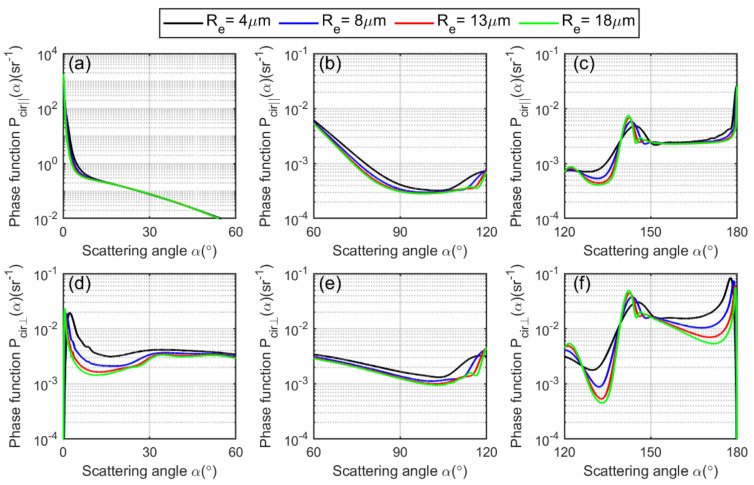
(**a**–**c**) show the circularly, parallelly polarized scattering-phase function of a spherical cloud droplet plotted versus scattering angle calculated by using Equation (19). (**d**–**f**) show the circularly, perpendicularly polarized scattering-phase function of a spherical cloud droplet plotted versus scattering angle calculated by using Equation (20). The scattering range at forward hemisphere is 0°–60°, at centered hemisphere it is 60°–120°, and at backward hemisphere it is 120°–180°.

These functions are plotted versus scattering angle in Figure 5.

**Figure 6:**
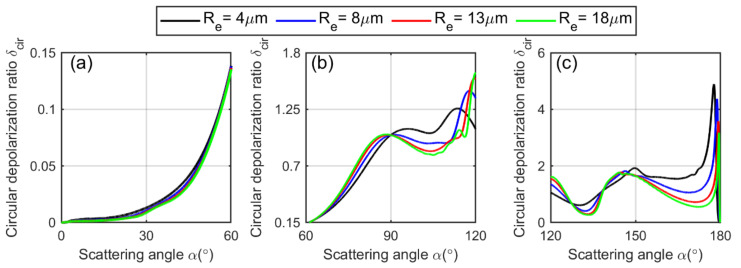
The theoretical circular depolarization ratio as a function of scattering angle of a single water cloud droplet (C1-cloud, γ=7,m=1.33) calculated by using Equation (21), (**a**) at forward hemisphere 0°–60°, (**b**) at centered hemisphere 60°–120° (**c**) and at backward hemisphere 120°–180°.

Using Mie theory, we can calculate the theoretical circular depolarization ratio per spherical water cloud droplet as a function of scattering angle by the equation [24]:

(21) $$\delta_{cir}(x,m,z,\alpha )=\frac{L_{1}(\alpha )+L_{2}(\alpha )\cos^{2}\alpha -2L_{3}(\alpha )\cos\alpha }{L_{1}(\alpha )+L_{2}(\alpha )\cos^{2}\alpha +2L_{3}(\alpha )\cos\alpha },$$

with $L_{1},L_{2}\;\mathrm{and}\;L_{3}$ given by Equation (15). It is plotted in Figure 6. Although the values of the circular depolarization ratio are much higher as compared to the values of linear depolarization ratio, they follow the same increasing and decreasing trends in the forward, centered and backward hemispheres. Therefore, the same conclusion can be derived as that of the linear depolarization ratio.

### 3.4. Volume Depolarization Ratio

The volume linear depolarization ratio of water clouds with certain depths for a linearly, parallelly polarized beam can be determined by:

(22) $$\delta_{lin}=\frac{I-Q}{I+Q}.$$

We can express Equation (22) in form of polarized signals as:

(23) $$\delta_{lin}=\frac{P_{⊥}}{P_{∥}},$$

where $P_{⊥}\;\mathrm{and}\;P_{∥}$ are the perpendicularly and parallelly polarized backscattered signals relative to the incident laser beam polarization. Equation (23) can be integrated from the cloud base $Z_{bot}$ to the cloud top $Z_{top}$ of water clouds to obtain the layer-integrated volume linear depolarization ratio $\Delta \delta_{lin}$ as:

(24) $$\Delta \delta_{lin}=\frac{\int_{z_{bot}}^{z_{top}}P_{⊥}(z)dz}{\int_{z_{bot}}^{z_{top}}P_{∥}(z)dz}.$$

Note that Equations (22)–(24) are also applicable if the incident beam is linearly, perpendicularly polarized and yield the same depolarization ratio. So, in our simulation, we keep our calculation limited to incident linearly, parallelly polarized beams. The volume circular depolarization ratio of water clouds in the case of left-handed, circularly polarized beams can be calculated as [24]:

(25) $$\delta_{cir}=\frac{I+V}{I-V}.$$

We can express Equation (25) in the form of polarized signals as [34]:

(26) $$\delta_{cir}=\frac{P_{⊥}}{P_{∥}}.$$

Similarly, we can express the layer-integrated volume circular depolarization ratio $\Delta \delta_{cir}$ using Equation (26) as:

(27) $$\Delta \delta_{cir}=\frac{\int_{z_{bot}}^{z_{top}}P_{⊥}(z)dz}{\int_{z_{bot}}^{z_{top}}P_{∥}(z)dz}.$$

where $P_{⊥}\;\mathrm{and}\;P_{∥}$ are the perpendicularly and parallelly polarized backscattered signals relevant to the incident beam polarization. Equations (25)–(27) are also applicable if the incident laser beam is right-handed and circularly polarized and yields the same depolarization ratio. So, we kept our calculation limited to the incident left-handed, circularly polarized beam.

## 4. Polarized Semianalytic Monte Carlo Simulation

**Figure 7:**
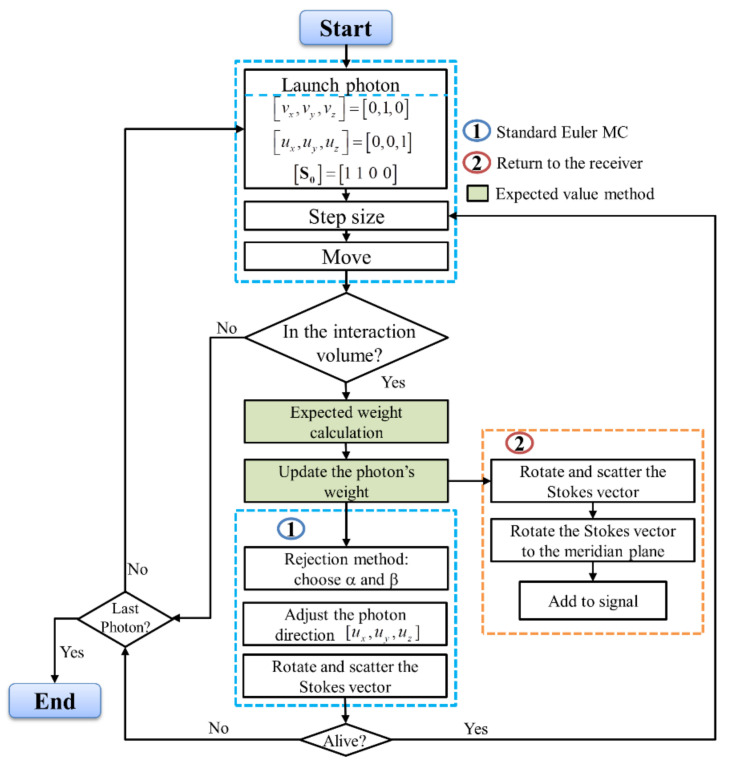
The flow chart of MC simulation procedure with both the standard and semianalytic features. The green highlighted cells show the steps specific to expected value method. The cells encircled by blue dotted lines tagged with “1” are the steps involved in the standard Euler MC method, while the cells encircled by orange dotted lines tagged with “2” are the steps of the photon returning to the receiver.

**Figure 8:**
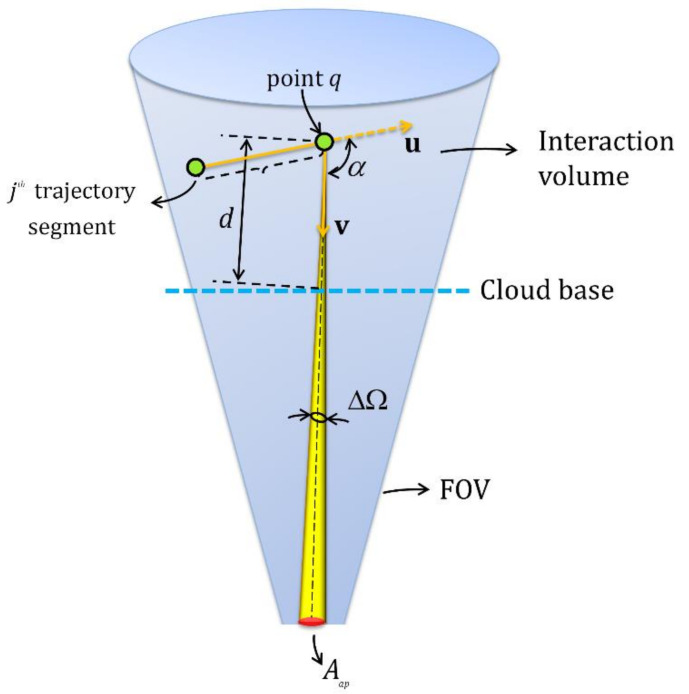
The schematic view of the scattering of fraction of photons weight at a point collected by the detector. $A_{ap}$ is the aperture defining the receiver field of view and ΔΩ corresponds to the angle for the FOV.

The multiple-scattering of polarized light in a medium can be modeled with the polarized Monte Carlo (PMC) simulation. The efficiency of the PMC simulation can be enhanced by increasing the number of launching photons. In effect, the signal quality is improved, thereby reducing the probability variance in the computation. However, it consumes a considerable amount of memory of the computer, limiting its computational speed. The alternative is to use the polarized semianalytic Monte Carlo (PSMC) approach, which combines both analytical and statistical methods to evaluate the simulated signal [29,53]. The schematic view of various steps involved in the PSMC method can be seen in Figure 7, which is well explained in [29]. In our PSMC simulation, we adopt the photon’s weight variance reduction method to speed up the convergence scheme [29]. The PSMC simulation is based on the expected weight of a portion of a photon’s packet which can be calculated by the combination of statistical and analytical algorithms and then returns to the detector without any interruption, as shown in Figure 8 [53].

The expected value of the fraction of photons weight collected by the detector upon scattering at a point can be written as [29]:

(28) $$E=\frac{P(\alpha )}{4\pi }\Delta \Omega \exp\left(-\int_{0}^{d}\alpha_{e}(z)dz\right)T_{m}^{2},$$

where P(α) is the scattering-phase function assumed to be constant over the small, solid angle ΔΩ and gives the fraction of the photon packet scattered into an element of solid angle ΔΩ about the direction α. The term $\exp(-\int_{0}^{d}\alpha_{e}(z)dz)$ is the probability that the photons scattered through an angle α will then be transmitted from the point to the air–cloud interface with no further interactions. The term $\alpha_{e}$ is of course the beam extinction coefficient in water clouds, d is the distance from the cloud base to interaction volume, and $T_{m}$ is the molecular transmission between the lidar and the cloud [29].

The laser beam is collimated and launched perpendicular to the scattering volume of the cloud. Since we are dealing with the polarized MC program, the polarization reference frame of the photon is defined and tracked with the Euler MC method. There are two other methods, the meridian plane and quaternion MC method, that can be used to track the polarization reference frame of the photon, which are well explained in [46]. The Euler MC method uses a triplet of unit vectors to track the polarization reference frame of the photon after every scattering event and needs less computation. Moreover, since for any scattering only one rotation of the reference frame is needed, the program should in principle be faster than other polarization tracking methods. An added advantage is that the propagation of the unit vectors is straightforward and easier to implement. In our implementation, only two vectors are rotated for every scattering event, v and u; the third unit vector is implicitly defined by the cross product of v and u and is only calculated when a photon reaches a boundary. The unit vectors are rotated using Euler angles by general transformation matrices [46].

Before the program begins, some additional steps need to be taken. The polarization reference frame of the field must be defined, and the Stokes vector describing the incident field of polarization must be declared. For our geometry of the beam, the initial position and direction of the photon can be defined by vectors v and u as [54]:

(29) $$v=\left[v_{x},v_{y},v_{z}\right]=\left[0,1,0\right]$$

and

(30) $$u=\left[u_{x},u_{y},u_{z}\right]=\left[0,0,1\right].$$

$u_{z}=1$ means that the photon is directed perpendicular to the cloud. The Stokes vector is used to define the initial polarization reference frame of the photon. For example, for a linearly, parallelly polarized incident beam, the Stokes vector is given by:

(31) $$S_{0}=\left[1\;1\;0\;0\right].$$

The photon is launched with the initial weight of 1 (W=1). The weight is discriminated by scattering as it propagates into the water clouds. The absorption ratio is quite a small parameter in water clouds in the visible range as compared to the scattering and is just neglected [35]. The direction cosines $\left[u_{x},u_{y},u_{z}\right]$ are used to specify the direction of each photon as [55]:

(32) $$\begin{array}{l} u_{x}=\sin\alpha \cos\beta ,\\ u_{y}=\sin\alpha \sin\beta ,\\ u_{z}=\cos\alpha , \end{array}$$

where α is the angle that the trajectory makes with respect to the z−axis, and β is the angle that the trajectory makes with the x−axis.

The propagation distance Δs covered by photon is determined by the probability density function using random numbers RND between 0 and 1, as:

(33) $$\Delta s=\frac{-\ln(RND)}{\alpha_{e}},$$

where $\alpha_{e}$ is the extinction coefficient of a water cloud. The photon position is updated after each scattering to a new position $\left[x^{\prime },y^{\prime },z^{\prime }\right]$ with the following equation [46]:

(34) $$\begin{array}{l} x^{\prime }=x+\Delta s\;u_{x},\\ y^{\prime }=y+\Delta s\;u_{y},\\ z^{\prime }=z+\Delta s\;u_{z}, \end{array}$$

where [x,y,z] and $\left[u_{x},u_{y},u_{z}\right]$ are the initial positions and direction cosines. The two angles, α (the angle of scattering) and β (the angle of rotation), are determined by the rejection method. The method requires pre-definition of the scattering-phase function given in Equation (7) [46].

A full description of how to implement the rejection method can be found in [27]. A short overview is presented here. The rejection method is used to generate random variables with a particular distribution. For unpolarized light, two random numbers are generated, $P_{rand}\;\mathrm{and}\;\alpha_{rand}$, in the range of 0–1 and 0−π. The angle $\alpha_{rand}$ is accepted as the new scattering angle if $P_{rand}\leq P(\alpha_{rand})$. When the angle $\alpha_{rand}$ is accepted, a similar procedure is adopted to calculate a new angle $\beta_{rand}$. For polarized light, three random numbers are generated: $P_{rand},\;\alpha_{rand}\;\mathrm{and}\;\beta_{rand}$. The angle β is uniformly distributed in the range of 0−2π. If $P_{rand}\leq P(\alpha_{rand},\beta_{rand})$, then both $\alpha_{rand}\;\mathrm{and}\;\beta_{rand}$ are accepted as the new angles. If $P_{rand}>P(\alpha_{rand})$, then $\alpha_{rand}$ and $P_{rand}$ are re-generated and the test is repeated [46]. Once the angles α and β are determined, the scattering process can be calculated. In the Euler MC method, a set of rotational angles are used to follow the Stokes vector reference frame called Euler angles [45]. The implementation of these angles is given in [46].

The Stokes vector reference frame is tracked with only two vectors v and u, and the rotations are implemented using the rotation matrix $R_{Euler}$. $R_{Euler}$ can be expressed using Rodriques’ formula [46]. In matrix form, it is given by [46]:

(35) $$R_{Euler}\left(k,\sigma \right)=\left[\begin{array}{ccc} k_{x}k_{x}v+c & k_{y}k_{x}v-k_{z}s & k_{z}k_{x}v+k_{y}s\\ k_{x}k_{y}v+k_{z}s & k_{y}k_{y}v+c & k_{y}k_{z}v-k_{x}s\\ k_{x}k_{z}v-k_{y}s & k_{y}k_{z}v+k_{x}s & k_{z}k_{z}v+c \end{array}\right],$$

where the unit vector $k=\left[k_{x},k_{y},k_{z}\right]$ is the rotation axis and c=cos(σ), s=sin(σ) and v=1−cos(σ), where σ is the angle of rotation. In the PSMC program, the vectors v and u are rotated twice in the following order. In the first rotation, the vector v is rotated about u through an angle β by multiplying v with the rotational matrix $R_{Euler}\left(u,\beta \right)$ (u remains unchanged in this process), while in the second rotation, the vector u is rotated about v through an angle α by multiplying u with the rotational matrix $R_{Euler}\left(v,\alpha \right)$ (v remains unchanged in this process).

Finally, the Stokes vector is adjusted for the two rotations. The Stokes vector is multiplied by the rotational matrix $R\left(\beta \right)$ and then by the scattering matrix $M\left(\alpha \right)$. These matrices are given by [46]:

(36) $$R(\beta )=\left[\begin{array}{cccc} 0 & 0 & 0 & 0\\ 0 & \cos(2\beta ) & \sin(2\beta ) & 0\\ 0 & -\sin(2\beta ) & \cos(2\beta ) & 0\\ 0 & 0 & 0 & 1 \end{array}\right]$$

and

(37) $$M(\alpha )=\left[\begin{array}{cccc} P_{11}(\alpha ) & P_{12}(\alpha ) & 0 & 0\\ P_{12}(\alpha ) & P_{11}(\alpha ) & 0 & 0\\ 0 & 0 & P_{33}(\alpha ) & P_{34}(\alpha )\\ 0 & 0 & -P_{34}(\alpha ) & P_{33}(\alpha ) \end{array}\right].$$

The new Stokes vector $S_{new}$ after scattering becomes:

(38) $$S_{new}=M\left(\alpha \right)R\left(\beta \right)S_{0}.$$

The life of a photon ends when the photon passes through a boundary or when its weight value falls below a threshold (typically 0.001) [54]. The first rotation is needed to return the Stokes vector to the meridian plane. To do this, w is reconstructed as the cross product of v and u, as [46]:

(39) w=v×u.

The angle ε needed to rotate the Stokes vector into a meridian plane is given by [46]:

(40) $$\begin{array}{l} \varepsilon =0\;\mathrm{when}\;v_{z}=0\;\mathrm{and}\;u_{z}=0,\\ \varepsilon =\tan^{-1}\left(\frac{v_{z}}{-w_{z}}\right)\;\mathrm{in}\;\mathrm{all}\;\mathrm{other}\;\mathrm{cases}. \end{array}$$

This rotation is about the direction of propagation of the photon, i.e., u−axis. This rotation of the polarization reference frame is in a meridian plane for which $v_{z}=0$. A second rotation by an angle φ about the z−axis will put the photon reference frame into the detector reference frame. All three reference axes w,v and u are affected by this rotation. Angle φ is calculated as [46]:

(41) $$\varphi =\tan^{-1}\left(\frac{u_{y}}{u_{x}}\right),$$

where φ will be positive for backscattering photons and negative for transmitted photons. The Stokes vector of the reflected photon is multiplied one final time by $R\left(\varphi \right)$. For a transmitted photon, the angle φ is [46]:

(42) $$\varphi =-\tan^{-1}\left(\frac{u_{y}}{u_{x}}\right).$$

Since the Stokes vectors can be superimposed, all photons traveling in the direction of a detector can be added once they have been rotated to the detector reference frame. Thus, the final Stokes vector is written as [46]:

(43) $$S_{final}=R(\varphi )R(\varepsilon )S_{0}.$$

These steps are repeated for every scattering event.

## 5. Mishchenko–Hovenier Relationship

**Figure 9:**
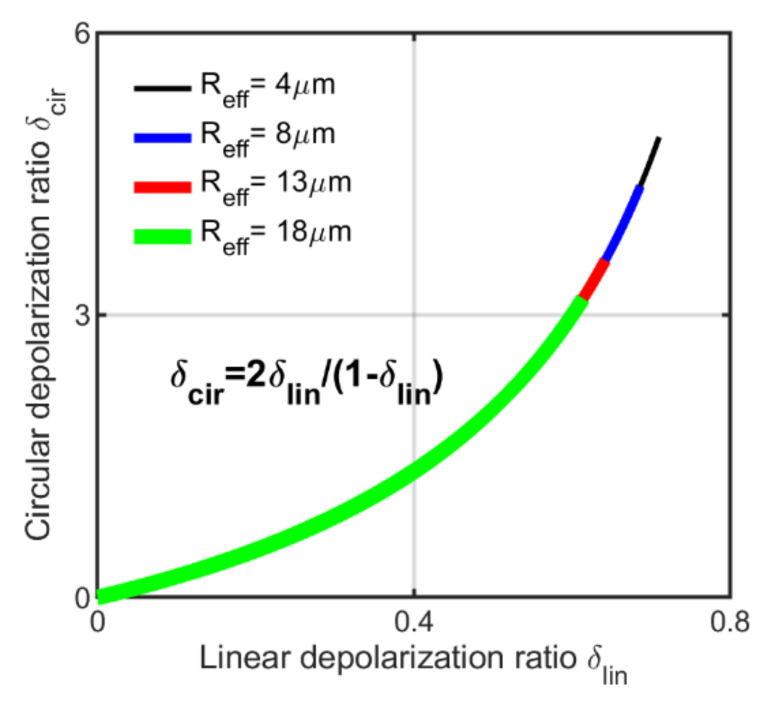
Theoretical circular depolarization ratio as a function of linear depolarization ratio known as Mishchenko–Hovenier relationship for four cloud droplet sizes distribution.

In light of Figure 4 and Figure 6, the theoretical curves of the circular depolarization ratio show the same increasing and decreasing trends as that of the linear depolarization ratio as a function of the scattering angles. Although the values of the circular depolarization ratio are 2 to 5 times higher than the linear depolarization ratio, their local maxima and minima coincide with each other, as depicted in the figures [24]. Based on these observations, a relationship between the two depolarization ratios is established by the authors in [2], known as the Mishchenko–Hovenier relationship. We can obtain this relationship using Equations (16) and (22) as [24]:

(44) $$\delta_{cir}\left(\alpha \right)=\frac{2\delta_{lin}\left(\alpha \right)}{1-\delta_{lin}\left(\alpha \right)},$$

where α is the scattering angle. We can see that the circular depolarization ratio is a monotonically increasing function of the linear depolarization ratio, as shown in Figure 9.

The relationship depends on the FOV of the receiver telescope and distance of the cloud from the lidar. In addition, the relationship holds for all particle size distribution [56]. It implies from Equation (44) that $\delta_{cir}$ increases with the particle size and refractive index if $\delta_{lin}$ increases and decreases if $\delta_{lin}$ decreases [2]. The authors in [2] showed that particles with a symmetry plane exhibit a similar relationship for a scattering angle of $180^{\circ }$. However, the author in [24] argued that the relationship could even be generalized for spherical particles as well if the depolarization ratio is averaged over the azimuthal angle for all scattering angles under multiple-scattering conditions [24]. He validated the relationship for water clouds using the calculation of double-scattering model as well as using the experimental results. However, in this article, we validate the Mishchenko–Hovenier relationship for all types of water cloud properties, including homogeneous, inhomogeneous and partially inhomogeneous, under multiple-scattering conditions using the PSMC simulation. The simulation results are presented in the next section.

## 6. Discussion

**Figure 10:**
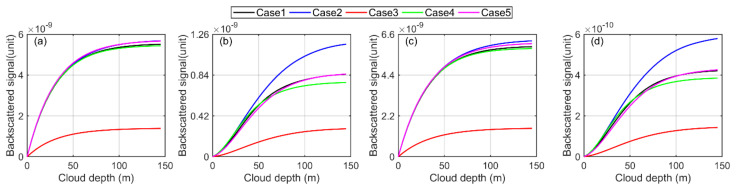
The simulated layer-integrated backscattered signal components obtained from the multiple-scattering of polarized beam in homogeneous water clouds using PSMC simulation (**a**,**b**) shows the circularly, parallelly and circularly, perpendicularly polarized backscattered signal components, whereas (**c**,**d**) show the linearly, parallelly and linearly, perpendicularly polarized backscattered signal components.

We begin our simulation by considering the homogeneous water cloud. The incident beam is first left-handed and circularly polarized, for which the Stokes vector is written as $S_{0}=\left[1\;0\;0\;-1\right]$. The scattered Stokes parameters are obtained from the simulation using the PSMC method. Using the scattered Stokes parameters, the layer-integrated backscattered signal components, polarized parallel and perpendicular, are obtained, as shown in Figure 10a,b. It is to be noted that when the laser beam is circularly polarized and incident on water cloud, the cloud droplet only changes its helicity without changing its intensity. This means that the water cloud droplets turn the beam polarization from left-handed into the right-handed circular and vice versa. We refer to the signal components as circularly, parallelly and circularly, perpendicularly polarized just to maintain a definition consistency between the linear and circular polarization beams. Therefore, the circular depolarization ratio is to be taken in the same sense as the linear depolarization ratio. The backscattered signals are layer-integrated in order to average the signal points from the bottom to the top of the cloud. Each signal belongs to each cloud case, tabulated in Table 1.

The simulation is repeated for each cloud case separately, and overall, ten backscattered signal components in pairs are obtained. It is to be noted that we consider the top layer of the water cloud as a complete boundary, and that is why the signals are truncated, just reaching the cloud top. In practice, the receiver channels of the polarization lidar should be sensitive to circular polarization to measure the backscattered and scattered circular polarized signals. In the case of linearly polarized (linear parallel), the Stokes vector is written as $S_{0}=\left[1\;1\;0\;0\right]$. The layer-integrated backscattered signal components, linearly, parallel and linearly, perpendicular, obtained can be seen in Figure 10c,d. If we compare the circularly, parallelly polarized and the linearly, parallelly polarized signal components in Figure 10a,c, we can argue that the signal intensities of circularly, parallelly polarized components are slightly less than that of linearly, parallelly polarized signal components. In contrast, the signal intensities of circularly, perpendicularly polarized components are significantly higher than that of linearly, parallelly polarized signal components. Our simulation revealed that no dramatic changes occurred when the incident left-handed, circularly polarized and linearly, parallelly polarized beams are changed into right-handed, circularly polarized and linearly, perpendicularly polarized beams, but only the exchange of signal components occurred. Therefore, the simulations of these two cases of incident beams are just ignored. From these observations, we can conclude that the measurement of water clouds is also feasible with circular polarization, and the depolarized signal is even more prominent.

**Figure 11:**
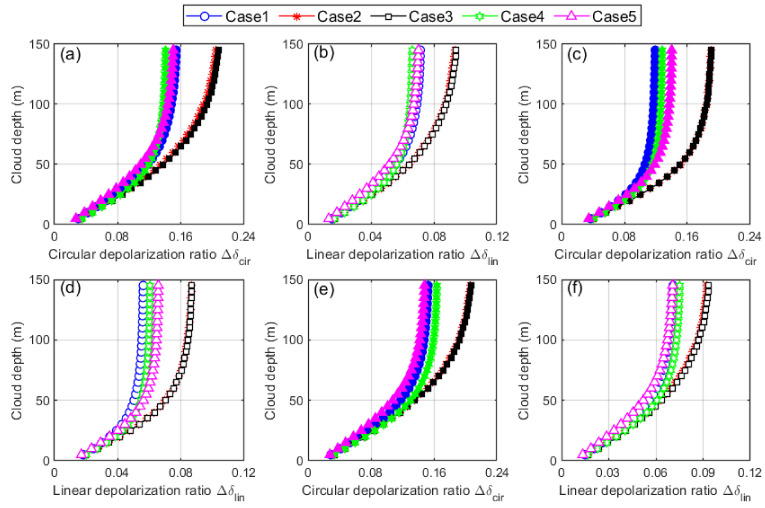
The layer-integrated volume depolarization ratio Δδ plotted versus height of the cloud including all five cloud cases of homogeneous (**a**,**b**), inhomogeneous (**c**,**d**), and partially inhomogeneous (**e**,**f**) water clouds. The Δδ curves with filled markers belong to the incident circularly polarized beam and with empty markers belong to the incident linearly polarized beam. The colors and shapes of the markers represent the cloud cases ranging from Case1 to Case5, as listed in Table 1.

Furthermore, the volume layer-integrated depolarization ratio Δδ for both circularly and linearly polarized beams are calculated using Equations (26) and (29). The Δδ is plotted versus the height of the cloud for all cloud cases of homogeneous, inhomogeneous and partially inhomogeneous water clouds, as shown in Figure 11. We can see that both the layer-integrated circular and linear depolarization ratios, $\Delta \delta_{cir}$ and $\Delta \delta_{lin}$, increase rapidly at the cloud base up to certain height and then gradually remain constant until the cloud top. All the curves of $\Delta \delta_{cir}$ and $\Delta \delta_{lin}$ show the similar increasing trend from the bottom to the top of the cloud. A slight variation in the curves is caused by the change in the cloud droplet effective radius and extinction coefficient. However, no scaling laws exist so far that truly define a relationship between the depolarization ratio and the cloud droplet effective radius and extinction coefficient [6]. Thus, both the $\Delta \delta_{cir}$ and $\Delta \delta_{lin}$ implicitly depend on the cloud effective radius and extinction coefficient. On the other side, the cloud Case2 and Case3 show high depolarization ratios as compared to other cases in both scenarios. This is because in cloud Case2, the value of the FOV is increased to be 1 and 2 mrad, causing an increase in the depolarization ratio because multiple-scattering is increased. Larger FOVs receive more multiple-scattered photons and thus causes high depolarization.

**Figure 12:**
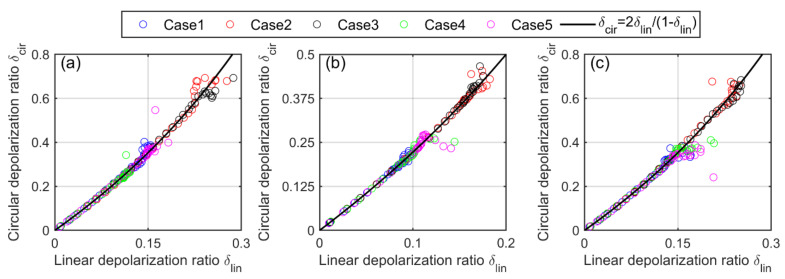
The validation of Mishchenko–Hovenier curve using fives cloud cases of (**a**) homogeneous, (**b**) inhomogeneous and (**c**) partially inhomogeneous water clouds via PSMC simulation.

The increase in depolarization ratio is also observed in the cloud Case3 by raising the height of the cloud bottom from 2005 m to 4010 m. The reason for this is that high clouds have larger footprints of the FOV, and thus, more multiple-scattered photons can participate in the backscattered lidar signal that causes an increase in the depolarization ratio. Thus, along with the cloud droplet effective radius and extinction coefficient, the height of the cloud bottom and the size of the FOV have significant impacts not only on the linear depolarization ratio but on circular depolarization ratio as well, which are evident from the figures. Finally, we validate the Mishchenko–Hovenier relationship (Equation (44), Figure 9) using the data of all five cloud cases of homogeneous, inhomogeneous and partially inhomogeneous water clouds. We can see in Figure 12 that our simulated data follow the same theoretical Mishchenko–Hovenier relationship between linear and circular depolarization ratios though the properties of each cloud case differ greatly. Thus, we can conclude that the Mishchenko–Hovenier relationship is valid for all kinds of water cloud properties under multiple-scattering conditions.

## 7. Conclusions

In this article, we discussed the polarization properties of homogeneous, inhomogeneous and partially inhomogeneous water clouds using a circular polarization lidar and compared it with that of a linear polarization lidar. We considered five different cloud cases in each type and carried out a simulation for each cloud case separately. We obtained the layer-integrated depolarization ratios under the multiple-scattering condition using the polarized semianalytic Monte Carlo (PSMC) method for both circular and linear polarization lidar. The comparison showed that both the linear and circular layer-integrated depolarization ratios increase rapidly at the cloud base and then gradually remain constant up to the top of the cloud. The comparison also reveals that similar trends persist in both the depolarization curves from the bottom to the top of the cloud in all cloud cases. However, as compared to the linear depolarization ratio, the values of the circular depolarization ratio were significantly high, which proved the high sensitivity of the circular polarization lidar measurement of water clouds.

We also observed that cloud effective radius and extinction coefficient have the same effect on both linear and circular depolarization ratios. In cloud Case2 and Case3, we increased the FOV from 1 mrad to 2 mrad and the height of the cloud bottom from 2005 m to 4010 m, and as a result, the same percent increase was observed in both circular and linear layer-integrated depolarization ratios. Finally, we validated the Mishchenko–Hovenier relationship for all five cloud cases of homogeneous, inhomogeneous and partially inhomogeneous water clouds. We proved that the Mishchenko–Hovenier relationship even holds for all kinds of water cloud properties under multiple-scattering conditions. Future work is required to study the feasibility of a circular polarization lidar in retrieving the microphysical properties of water clouds.

## Figures and Tables

**Table 1:** The input parameters of water clouds and lidars required for simulations.

Cloud Type		Cloud Case	FOV (mard)	Cloud Base Height (m)	Cloud Depth (m)	Effective Radius *R_e_* (μm)		Extinction Coefficient *α_e_* (m^−1^)	
Homogeneous water cloud		Case1	1	2005	150	9		0.02	
		Case2	2	2005	150	9		0.02	
		Case3	1	4010	150	9		0.02	
		Case4	1	2005	150	9		0.01	
		Case5	1	2005	150	12		0.02	
Inhomogeneous water cloud		Case1	1	2005	150	4, 5, …, 23		$\alpha_{e1},\alpha_{e2},\ldots ,\alpha_{e20}$	
		Case2	2	2005	150	4, 5, …, 23		$\alpha_{e1},\alpha_{e2},\ldots ,\alpha_{e20}$	
		Case3	1	4010	150	4, 5, …, 23		$\alpha_{e1},\alpha_{e2},\ldots ,\alpha_{e20}$	
		Case4	1	2005	150	4, 5, …, 23		$\alpha_{e21},\alpha_{e22},\ldots ,\alpha_{e40}$	
		Case5	1	2005	150	6, 7, …, 25		$\alpha_{e1},\alpha_{e2},\ldots ,\alpha_{e20}$	
Partially inhomogeneous water cloud		Case1	1	2005	150	9		$\alpha_{e1},\alpha_{e2},\ldots ,\alpha_{e20}$	
		Case2	2	2005	150	9		$\alpha_{e1},\alpha_{e2},\ldots ,\alpha_{e20}$	
		Case3	1	4010	150	9		$\alpha_{e1},\alpha_{e2},\ldots ,\alpha_{e20}$	
		Case4	1	2005	150	9		$\alpha_{e21},\alpha_{e22},\ldots ,\alpha_{e40}$	
		Case5	1	2005	150	12		$\alpha_{e1},\alpha_{e2},\ldots ,\alpha_{e20}$	
$\alpha_{e1}$	0.0200	$\alpha_{e2}$	0.0225	$\alpha_{e3}$	0.0252	$\alpha_{e4}$	0.0278	$\alpha_{e5}$	0.0295
$\alpha_{e6}$	0.0313	$\alpha_{e7}$	0.0333	$\alpha_{e8}$	0.0364	$\alpha_{e9}$	0.0385	$\alpha_{e10}$	0.0397
$\alpha_{e11}$	0.0411	$\alpha_{e12}$	0.0432	$\alpha_{e13}$	0.0465	$\alpha_{e14}$	0.0484	$\alpha_{e15}$	0.0498
$\alpha_{e16}$	0.0519	$\alpha_{e17}$	0.0543	$\alpha_{e18}$	0.0576	$\alpha_{e19}$	0.0587	$\alpha_{e20}$	0.0600
$\alpha_{e21}$	0.0210	$\alpha_{e22}$	0.0236	$\alpha_{e23}$	0.0249	$\alpha_{e24}$	0.0278	$\alpha_{e25}$	0.0292
$\alpha_{e26}$	0.0310	$\alpha_{e27}$	0.0330	$\alpha_{e28}$	0.0350	$\alpha_{e29}$	0.0370	$\alpha_{e30}$	0.0395
$\alpha_{e31}$	0.0408	$\alpha_{e32}$	0.0432	$\alpha_{e33}$	0.0458	$\alpha_{e34}$	0.0481	$\alpha_{e35}$	0.0497
$\alpha_{e36}$	0.0510	$\alpha_{e37}$	0.0540	$\alpha_{e38}$	0.0570	$\alpha_{e39}$	0.0590	$\alpha_{e40}$	0.0600
